# Predicting Receiver Characteristics without Sensors in an LC–LC Tuned Wireless Power Transfer System Using Machine Learning

**DOI:** 10.3390/s24020501

**Published:** 2024-01-13

**Authors:** Minhyuk Kim, Wend Yam Ella Flore Niada, Sangwook Park

**Affiliations:** 1 EM Environment R&D Department, Korea Automotive Technology Institute, Cheonan 31214, Republic of Korea; mhkim@katech.re.kr; 2 Department of Electronic Engineering, Daegu University, Gyeongsan 38453, Republic of Korea; niadaellaflore@daegu.ac.kr; 3 Department of Electronic Engineering, Soonchunhyang University, Asan 31538, Republic of Korea

**Keywords:** wireless power transfer, impedance matching, machine learning, load resistance estimation, coupling coefficient estimation

## Abstract

Improvement of wireless power transfer (WPT) systems is necessary to tackle issues of power transfer efficiency, high costs due to sensor and communication requirements between the transmitter (Tx) and receiver (Rx), and maintenance problems. Analytical techniques and hardware-based synchronization research for Rx-sensorless WPT may not always have been available or accurate. To address these limitations, researchers have recently employed machine learning (ML) to improve efficiency and accuracy. The objective of this work was to replace Tx–Rx communication with ML, utilizing Tx-side parameters to predict the load and coupling coefficients on an LC–LC tuned WPT system. Based on current and voltage features collected on the Tx-side for various load and coupling coefficient values, we developed two models for each load and coupling prediction. This study demonstrated that the extra trees regressor effectively predicted the characteristics of LC–LC tuned WPT systems, with coefficients of determination of 0.967 and 0.996 for load and coupling, respectively. Additionally, the mean absolute percentage errors were 0.11% and 0.017%.

## 1. Introduction

Wireless power transfer (WPT) is being explored as a substitute for traditional power transmission methods in various industries, such as biomedical implants [1], wirelessly powered sensors [2], and electric vehicles [3,4]. Its benefits include cost savings through reduced use of wires and cables, improved security, increased mobility, ease of installation, and low maintenance. Resonant WPT, developed at MIT in 2007, is one of the several WPT techniques implemented for medium-range WPT. Communication is a crucial element in optimizing power transmission in such a system due to transmitter (Tx) and receiver (Rx) separation. Sensors are utilized for communication to verify position and alignment, but if a separate sensor and communication circuit on the Rx side can be separated from the circuit for WPT, the manufacturing cost of the Rx side can be reduced in the overall cost. Safety costs can also be reduced by not consuming standby power of the device being charged by the Rx. Furthermore, sensors may require protection against extreme temperatures as they can be fragile, and their measurements may also be compromised due to interference from other sensors or electromagnetic fields.

Research about Rx-side sensorless WPT systems has been undertaken in [5,6,7,8,9,10,11,12,13,14,15]. The study in [15] proposes a machine learning (ML) assisted method that estimates the power delivered to the Rx by using only measurements at the Tx-side. A method is proposed based on the delivered power estimation to identify if the system efficiency is too low, and the Tx should be turned off. This activation control method can be useful in multi-Tx WPT systems. One of the main challenges in WPT devices is the performance degradation when the receiver’s position and characteristics vary. Therefore, monitoring the load resistance and receiver position is essential for optimizing power transfer. Additionally, an ML method is proposed to estimate the load resistance and coupling coefficient [15]. This method accurately predicts the characteristics of an LCC-Series tuned WPT system by analyzing only the measured root-mean-square and harmonic contents of the input current. The LCC matching circuit is a commonly utilized circuit in WPT systems due to its high efficiency, ability to tolerate misalignment, and zero voltage switching. It is primarily used for high frequency and electric vehicle charging applications. WPT systems use different matching networks with different characteristics, which have different effects on the whole circuit. Therefore, conducting a study focused on different matching networks with different inputs in the ML can provide additional data for using ML in WPT to predict load and coupling using only Tx-side parameters.

Our study proposes estimating the load and coupling coefficient [15,16,17] on an LC–LC matching-based WPT system using artificial intelligence with only Tx-side parameters. During the charging process, the load value fluctuates [18], affecting the transferred power. The coupling coefficient evaluates the strength of the coupling between the Tx and Rx and impacts the power transfer efficiency (PTE) [19,20]. The LC–LC circuit is a symmetrical matching network that offers a resonance advantage independent of the load [21]. This eliminates the need for an adaptive impedance matching topology to address power loss due to mismatches as the load independence varies during charging. LC–LC further provides a load-independent, constant current. LC matching networks are frequently used in WPT applications including electric vehicle charging systems [21,22,23,24,25]. In our study, we selected input current and Tx-side matching circuit capacitor voltage parameters. This decision was motivated by the goal of diversifying the input data and observing how these features impact the predicted values.

In this paper, we constructed the equivalent circuit of an LC–LC tuned WPT system to analyze the WPT circuit. Then, using the equations obtained from the analysis, we collected the dataset required for ML through calculation, by extracting the features from the input current and the Tx-side capacitor voltage. With the obtained dataset, we could obtain the feature importance, and the estimated results using various regression algorithms.

## 2. LC–LC WPT Circuit Analysis

The LC matching network refers to a circuit designed to provide using an inductor and a capacitor. However, when the Tx/Rx coil is also taken into consideration, it may be referred to as LCL. Hence, in some other studies, LCL is used when referring to the LC network.

**Figure 1:**
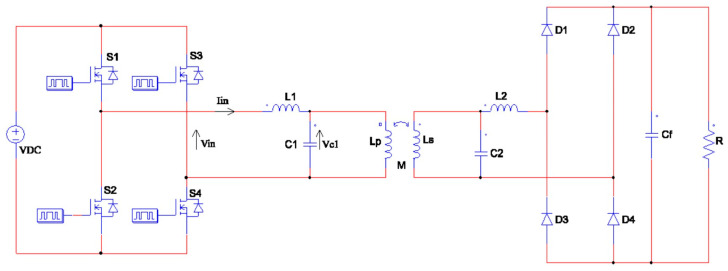
WPT LC–LC matching circuit.

Our circuit, depicted in Figure 1, was constructed like any other WPT system. On the Tx side, the system was comprised of a DC source, followed by an inverter made up of four switching transistors (*S*_1_–*S*_4_) arranged in a bridge configuration. This inverter served to convert the DC to high frequency AC. Next, the Tx-side matching circuit consisted of an inductor (*L*_1_) and capacitor (*C*_1_) in series. The circuit continued with the Tx coil (*L_p_*) and the Rx coil (*L_s_*). On the Rx side, the circuit followed through with the Rx-side matching circuit consisting of capacitor *C*_2_ and inductor *L*_2_ after the Rx coil. The rectifier following the matching circuit consists of a full bridge of diodes (*D*_1_−*D*_4_) and a filter capacitor (*C_f_*), which both contribute to the AC–DC conversion. *R* denotes the battery that is being charged.

### Equivalent Circuit Theoretical Analysis

**Figure 2:**
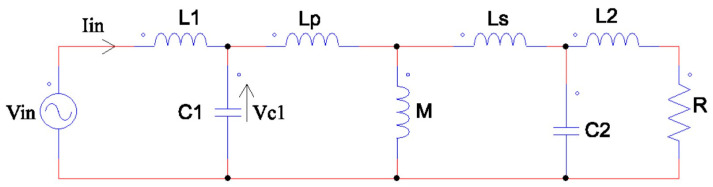
Equivalent circuit.

The equivalent circuit can be simplified as in [15,26], as shown in Figure 2. The inverter circuit can be reduced to a sinusoidal voltage source (1) and the rectifier circuit, in combination with the load, provides an equivalent load impedance (2). The voltage is then transmitted through the LC resonant circuit model, from the primary to the secondary, and eventually to the load.

(1) $$V_{in}=\frac{4V_{DC}}{\pi }\sin(\omega_{0}t)$$

where *V_in_* represents the AC voltage source of the circuit, and $\omega_{0}=2\pi f,$ where *f* is the resonant frequency.

(2) $$R_{eq}=\frac{\pi^{2}R}{8}$$

*R_eq_* stands for the equivalent load value. Our analysis, which employed Kirchhoff’s laws, yielded the following results:

(3a) $$V_{in}-Z_{L1}I_{in}-Z_{C1}{(I}_{in}-I_{p})=0$$

(3b) $$Z_{C1}{(I}_{in}-I_{p})-Z_{Lp}I_{p}-Z_{M}{(I}_{p}-I_{s})=0$$

(3c) $$Z_{M}{(I}_{p}-I_{s})-Z_{Ls}I_{s}-Z_{C2}{(I}_{s}-I_{2})=0$$

(3d) $$Z_{C2}{(I}_{s}-I_{2})-(Z_{L2}+R_{eq)}I_{2}=0$$

where *Z_L_*_1_, *Z_C_*_1_, *Z_Lp_*, *Z_M_*, *Z_Ls_*, *Z_L_*_2_ and *Z_C_*_2_ represent, respectively, the impedance at the resonant frequency of *L*_1_, *C*_1_, *L_p_*, mutual inductance (*M*), *L_s_*, *L*_2_ and *C*_2_. And, *I_in_*, *I_p_*, *Is* and *I*_2_ represent input current and current flowing through *L_p_*, *L_s_* and *L*_2_.

(4a) $$V_{in}=Z_{11}I_{in}+Z_{12}I_{2}$$

(4b) $$V_{s}=Z_{21}I_{in}+Z_{22}I_{2}$$

where *Z*_11_, *Z*_12_, *Z*_21_, *Z*_22_ represent the expression of input–output impedance based on the input and output currents. Equation (5) depicts the expression of the mutual inductance which is related to the coupling coefficient. The variation of k will proportionally affect the mutual inductance value.

(5) $$M=k\sqrt{L_{p}L_{s}}$$

The complete expressions of *Z*_11_, *Z*_12_, *Z*_21_, *Z*_22_ (6) and (7) show that these impedances are affected by the mutual inductance and the load value.

(6a) $$Z_{11}=Z_{L1}+Z_{c1}$$

(6b) $$Z_{12}=-\frac{\left(\frac{j}{C2*\omega_{0}}-C2*\omega_{0}*\left(R+L2*\omega_{0}*j-\frac{j}{C2*\omega_{0}}\right)*\left(Ls*\omega_{0}*j+M*\omega_{0}*j-\frac{j}{C2*\omega_{0}}\right)*j\right)}{C1*M*{\omega_{0}}^{2}}$$

(6c) $$Z_{21}=-\frac{M*j}{A*B*C1*C2*\omega_{0}}$$

(6d) $$Z_{22}=-L2*\omega_{0}*j+\frac{j}{C2*\omega_{0}}-\frac{1}{B*C2^{2}*{\omega_{0}}^{2}}$$

where

(7a) $$A=Lp*\omega_{0}*j+M*\omega_{0}*j-\frac{j}{C1*\omega_{0}}$$

(7b) $$B=Ls*\omega_{0}*j+M*\omega_{0}*j-\frac{j}{C2*\omega_{0}}+\frac{M^{2}*{\omega_{0}}^{2}}{A}$$

Through the analysis of impedance Equation (6), the primary and secondary impedance resistances were adjusted to attain the resonant state for power transmission. For *Im(Z*_11_*)* and *Im(*Z_22_*)* both equal to 0 the actual resonant point *ω*_0_ was reached [27]:

(8) $$\omega_{0}=\sqrt{\frac{1}{L_{1}C_{1}}+\frac{1}{L_{p}C_{1}}}=\sqrt{\frac{1}{L_{2}C_{2}}+\frac{1}{L_{s}C_{2}}}$$

Equation (8) represents the relationship between the circuit elements to achieve resonance. The parameters *I_in_* (9) and *V_C_*_1_ (10) were used for ML training. The coupling value was varied to affect the mutual inductance according to Equation (5) in order to simulate different coupling states between the Tx and Rx. We also varied the load value within a certain range for each coupling value to imitate battery charging, accounting for load fluctuations during the charging process. To gather information, we modified the coupling and load variables, which affected the voltage and current on the transmission side.

(9) $$I_{in}=\frac{V_{in}}{Z_{in}}$$

(10) $$V_{C1}=V_{in}-Z_{L1}I_{in}$$

**Figure 3:**
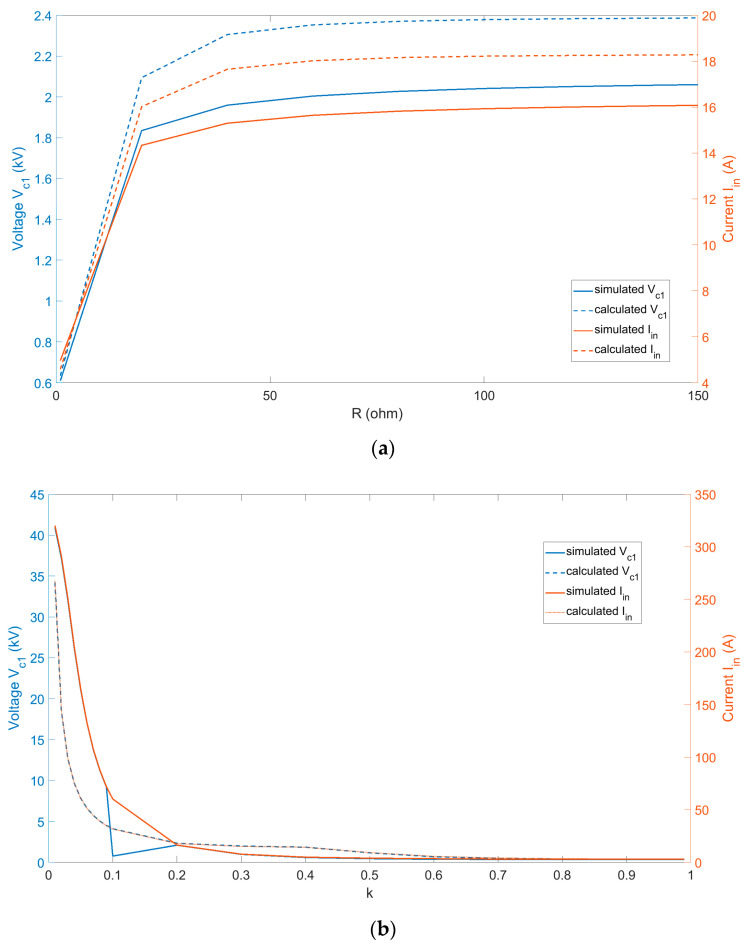
Comparison of measured and calculated values of *V_c_*_1_ and *I_in_* (**a**) Load (**b**) Coupling coefficient *k*.

In this paper, we designed a WPT system to charge mobile devices and low-power drones. The system is capable of transferring tens to hundreds of watts of power at a frequency of 6.78 MHz. Figure 3 presents a comparison between the simulated (PSIM 9.0 software environment) and theoretically calculated RMS values of *I_in_* and *V_C_*_1_, which varied depending on the load and coupling coefficient. Graphs depicting the load variation in Figure 3a show that while the simulated and calculated values had different numerical values, they exhibited the same shape. This was the same for the variation in the coupling factor in Figure 3b, where the graphs also converged in a similar manner. For *k* ≥ 0.6, the graphs combined as one.

Since our matching network was symmetrical, the elements on the Tx and Rx sides were identical in the case of Tx coil = Rx coil, which was applicable to our situation. After performing the calculation, the values in Table 1 were obtained. To achieve maximum data harvesting, *I_in_* and *V_C_*_1_ were calculated for the load values ranging from 1 to 150 and with coupling between 0.01 and 1, resulting in 15,000 data points. The structure of the data is illustrated in Table 2. For low coupling coefficient values, the voltage *V_C_*_1_ was in the kilovolt range due to poor coupling between the Tx and Rx. As the coupling coefficient increased, the voltage *V_C_*_1_ decreased, making the system more stable. For the load, the input current decreased while the voltage *V_C_*_1_ increased, which can be explained by circuit theory.

## 3. Machine Learning Approach

ML is a field of computer science and artificial intelligence that focuses on using data and algorithms to enable computers to learn without explicit programming. Our objective was to train ML models capable of predicting load and coupling coefficient values based on primary side voltage and current features. The data obtained from the calculations was used to extract features from signals.

### 3.1. Data Collection

In the ML model training described in [15], features used consisted of the input current’s RMS, magnitude, and phase of harmonics one and three. In our own study, we first calculated the current *I_in_* and voltage *V_C_*_1_ using Matlab code, then extracted features via different Matlab commands. Both *I_in_* and *V_C_*_1_ are highly responsive to changes in the load and coupling coefficient. Here are the features we extracted on time domain:

- RMS of *I_in_* and voltage *V_C_*_1_ (RMS(*I_in_*) and RMS(*V_C_*_1_));
- MAX of *I_in_* and voltage *V_C_*_1_ (MAX(*I_in_*) and MAX(*V_C_*_1_)).

Using the Fourier transform on the time domain signals, we switched to the frequency domain. In the frequency domain, we extracted spectrum amplitude and phase features on the resonant frequency:

- Amplitude of *I_in_* and voltage *V_C_*_1_ (*I_in-amp_* and *V_C_*_1*-amp*_);
- Phase of *I_in_* and voltage *V_C_*_1_ (*I_in-ph_* and *V_C_*_1*-ph*_);
- Phase difference between *I_in_* and voltage *V_C_*_1_ (phdiff(*I_in_* − *V_C_*_1_)).

**Figure 4:**
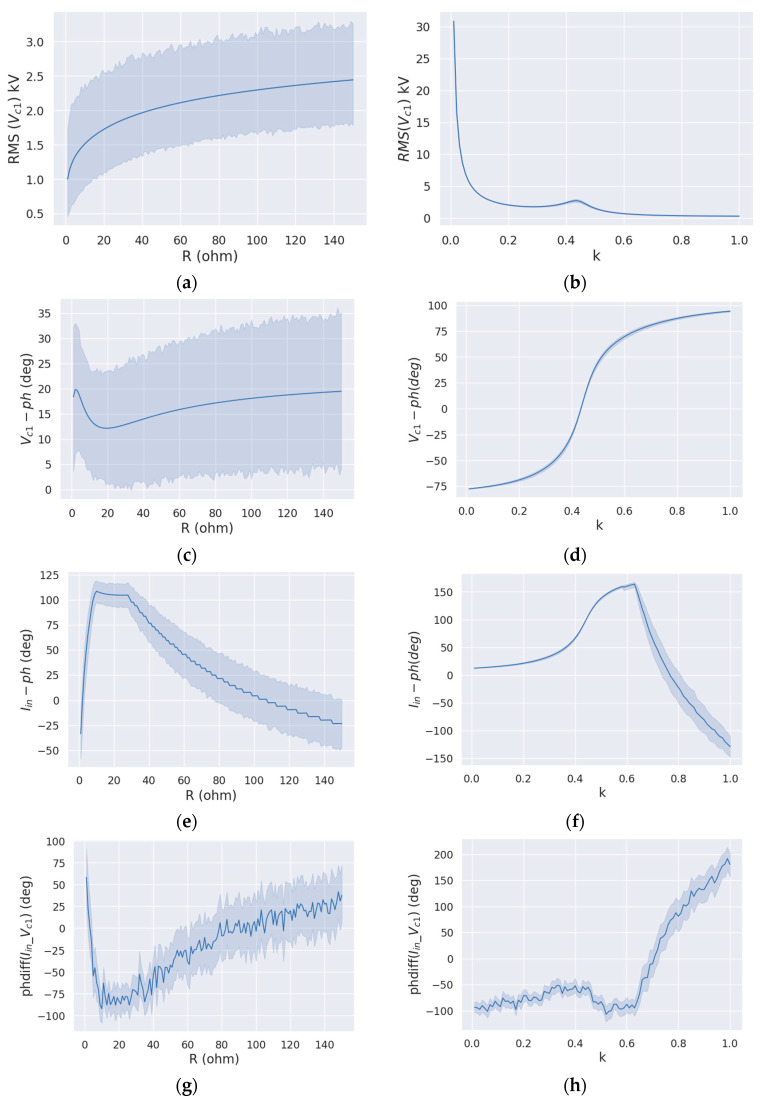
Graphs of the variation of extracted features based on load and coupling. (**a**) RMS of *V_C_*_1_ vs. load; (**b**) RMS of *V_C_*_1_ vs. coupling; (**c**) phase of *V_C_*_1_ spectrum vs. load; (**d**) phase of *V_C_*_1_ spectrum vs. coupling; (**e**) phase of *I_in_* spectrum vs. load; (**f**) phase of *I_in_* spectrum vs. coupling; (**g**) phase difference between *I_in_* and *V_C_*_1_ vs. load; (**h**) phase difference between *I_in_* and *V_C_*_1_ vs. coupling.

Figure 4 depicts the changes in various characteristics as load and coupling are adjusted. Figure 4a displays the graph of the RMS of *V_C_*_1_ versus the load. With increasing load values, the RMS of *V_C_*_1_ also increased. The graphs of the RMS of (*I_in_*, amplitude of *I_in_* and *V_C_*_1_), MAX of (*I_in_*, *V_C_*_1_, amplitude of *I_in_*, and *V_C_*_1_) versus load exhibited a similar shape to the graph in Figure 4a due to the proportional relationship between *I_in_* and *V_C_*_1_, following Equations (9) and (10). Figure 4b shows the graph of RMS of *V_C_*_1_ versus coupling. As the value of *k* increased, the RMS of *V_C_*_1_ decreased. The presented graphs demonstrate the relationship of the RMS and MAX values of (*I_in_*, amplitude of *I_in_*, and *V_C_*_1_) versus the coupling factor, showing the same shape as the graph presented in Figure 4b. In Figure 4c, the graph displays the *V_C_*_1_ spectrum phase versus the load, which demonstrated a significant decrease, followed by a slight increase. Similarly, in Figure 4d, as the coupling factor increased, the phase angle also increased. As shown in Figure 4e, there was a slight increase and decrease in the phase of the input current as the load value increased. Figure 4f displays the *V_C_*_1_ spectrum phase changing significantly by coupling, resulting in increase and deceleration. Figure 4g,h display graphs that were unstable, which may suggest that the phase difference feature had the least impact on the prediction of load and coupling. After examining all the graphs, it is apparent that the phase parameter displayed varying shapes while the other parameters featured consistent or erratic graphs. Therefore, it is possible to anticipate that the phase parameter may play a major or minor role in determining the load and coupling factor.

### 3.2. Model Evaluators

- The coefficient of determination (*R*^2^) in a regression model is a statistical measure that calculates the portion of the variation in the dependent variable that can be attributed to the independent variable. As such, it indicates the degree to which the data align with the regression model. When the coefficient of determination is 0, the model accounts for none of the variation in the response data around the mean. Conversely, when the measure is 1, the model accounts for all the variation in the response data around the mean. Therefore, a coefficient of determination value of approximately 1 signifies that the model provides a strong estimate. The formula to calculate the *R*^2^ is as follows:

(11) $$R^{2}={\left(\frac{\sum {(x}_{i}-\bar{x})(y_{i}-\bar{y})}{\sqrt{\sum {\left(x_{i}-\bar{x}\right)}^{2}\sum {\left(y_{i}-\bar{y}\right)}^{2}}}\right)}^{2}$$

$x_{i}$ = values of the input variable;

$\bar{x}$ = mean of the value of x-variable;

$y_{i}$ = values of the output variable;

$\bar{y}$ = mean of the value of y-variable;

- *MAPE* (mean absolute percentage error) is a metric in regression machine learning representing the percentage error between the predicted and actual value. A low *MAPE* value corresponds to a well-trained model. The formula to calculate the *MAPE* value is as follows:

(12) $$MAPE=\frac{100\%}{n}\sum \left|\frac{y-\hat{y}}{y}\right|$$

y = actual value;

$\hat{y}$ = predicted value;

n = number of predictions.

### 3.3. Feature Importances

**Figure 5:**
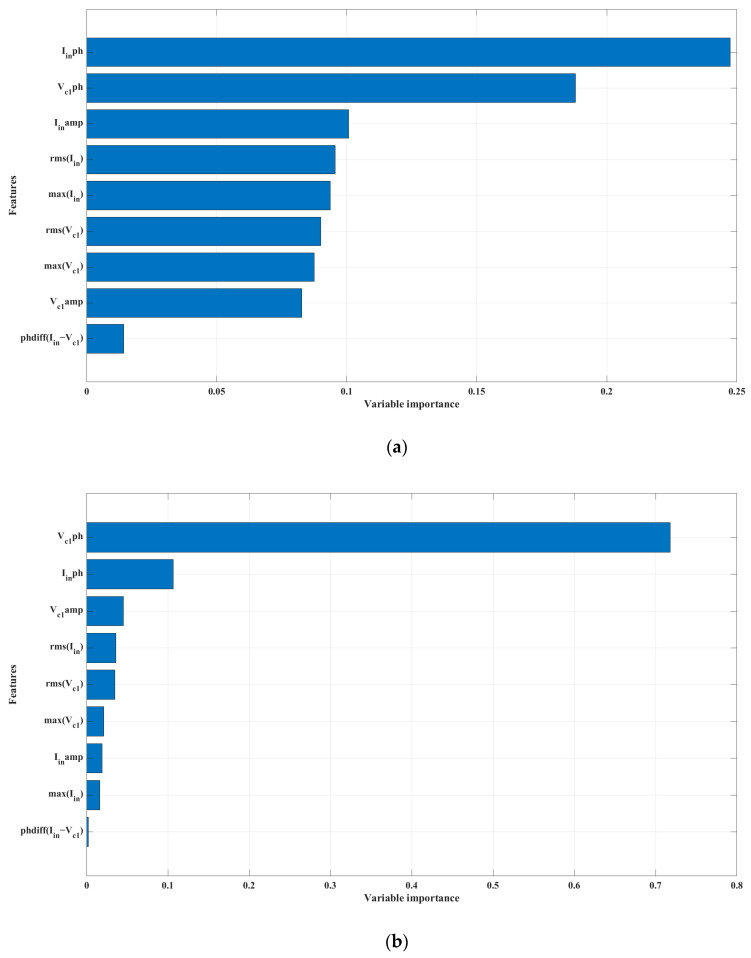
Feature importance on (**a**) load prediction (**b**) coupling prediction.

Based on Figure 5, the input current phase was essential for load estimation, whereas the voltage *V_C_*_1_ phase was vital for coupling prediction. Additionally, the other features, aside from the *V_C_*_1_ voltage phase, were insignificantly relevant for the prediction of the coupling factor, as illustrated in Figure 5b. The most significant feature for load prediction as depicted in Figure 5a, had a comparatively low value. However, the difference between this feature and the others was not substantial, which significantly demonstrated that most variables had a noteworthy impact. Comprehending the importance of variables enables the selection of suitable inputs that will promise the most precise predictions. Removing low-ranking features will reduce memory usage, computational costs, boost model accuracy, and prevent overfitting.

## 4. Discussion

**Figure 6:**
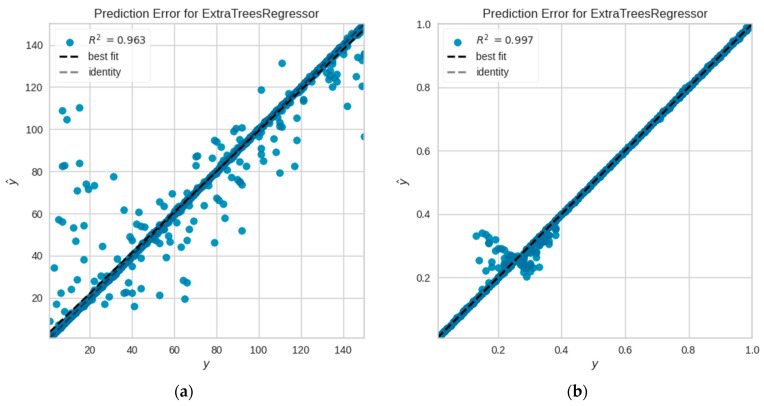
Prediction error for extra trees regressor on (**a**) load (**b**) coupling.

Table 3 presents the *R^2^* and *MAPE* values for the five best-trained model algorithms. The extra trees regressor algorithm produced the lowest *MAPE* value for both predictions. The accuracy of the load prediction model was approximately 99.89%, and the coupling prediction’s accuracy was around 99.98%. Consistent with the findings in [15], the coupling prediction was more accurate than the load prediction. The observed discrepancy is illustrated in Figure 6, where the predicted value which was $\hat{y}$, was compared to the actual value y. Specifically, in Figure 6b, which represents the coupling, the points cluster around the first bisector, while for the load in Figure 6a, some values deviated from the first bisector.

Our decision to approach WPT characteristic estimation for a LC–LC tuned WPT system has resulted in significant improvements, utilizing ML. The phase of *I_in_* and *V_C_*_1_ is a crucial indicator for predicting both load and coupling. Furthermore, our use of only Tx-side parameters makes implementing a communication circuit between Tx and Rx unnecessary. However, our study is purely theoretical; thus, the next step would be to conduct an experimental study and extend it to a multi-Tx WPT system as well.

## 5. Conclusions

In WPT, it is crucial to predict the load and coupling coefficients of the receiver without relying on sensors or communication between the transmitter and receiver. This is important to reduce the cost of WPT systems and minimize standby power consumption. This work extends the research on LCC-tuned WPT in Rx-sensorless WPT using ML. ML is gaining prominence due to its ability to overcome many existing limitations. The goal is to predict the receiver characteristics of LC–LC tuned WPT, which provides resonance benefits independent of load. In particular, the Tx-side parameters of input current and voltage of the matching network capacitor were used to predict the load and coupling coefficients. Relevant features were extracted, indicating that the phase of Iin and VC1 scored highest in importance. The extra trees regressor demonstrated the highest accuracy rates of 99.89% and 99.98% for the load and coupling factor, respectively. The findings of our study could lead to the prediction of multi-Tx WPT system characteristics via ML in the future.

## Figures and Tables

**Table 1:** Parameter values.

Frequency	Input Voltage	*L*_1_, *L*_2_	*C*_1_, *C*_2_	*L_p_, L_s_*	*R_L_*	*K*
6.78 MHz	50 V	3 μH	206.6 pF	24 μH	1:1:150 ^1^	0.01:0.01:1 ^2^

^1^ Load value goes from 1 to 150 with 1ohm step. ^2^ Coupling factor value goes from 0.01 to 1 with 0.01 step.

**Table 2:** Dataset structure.

Parameters	Min.	Max.	Step
Load	1	150	1
Coupling coefficient	0.01	1	0.01
Number of data sets	15,000		

**Table 3:** Data structure.

Algorithms	Evaluators	Load	Coupling
Random Forest	*R* ^2^	0.954	0.996
	*MAPE*	0.135%	0.02%
Decision Tree	*R* ^2^	0.921	0.993
	*MAPE*	0.141%	0.021%
Extra Trees Regressor	*R* ^2^	0.969	0.996
	*MAPE*	0.104%	0.017%
XGBoost	*R* ^2^	0.951	0.995
	*MAPE*	0.179%	0.0258%
Light Gradient Boost	*R* ^2^	0.94	0.996
	*MAPE*	0.2%	0.0296%

## Data Availability

Data is available upon request.
